# Valproic acid promotes radiosensitization in meningioma stem-like cells

**DOI:** 10.18632/oncotarget.3692

**Published:** 2015-03-29

**Authors:** Hsin-Ying Clair Chiou, Wen-Kuo Lai, Li-Chun Huang, Shih-Ming Huang, Sheau-Huei Chueh, Hsin-I Ma, Dueng-Yuan Hueng

**Affiliations:** ^1^ Department of Neurological Surgery, Tri-Service General Hospital, National Defense Medical Center, Taipei, Taiwan, R.O.C.; ^2^ Department of Biochemistry, National Defense Medical Center, Taipei, Taiwan, R.O.C.

**Keywords:** meningioma, tumor stem-like cells, radiosensitization, valproic acid, Oct4

## Abstract

Although meningioma stem-like cells have been isolated and characterized, their therapeutic targeting remains a challenge. Meningioma sphere cells (MgSCs) with cancer stem cells properties show chemo- and radioresistance in comparison with meningioma adherent cells (MgACs). We tested the effect of valproic acid (VPA), a commonly used anti-epileptic drug, which passes the blood brain barrier, on cultured MgSCs. VPA reduced the viability of MgSCs and MgACs. In MgSCs, treatment with VPA increased radio-sensitivity, expression of p-cdc2, p-H2AX and cleaved caspase-3 and PARP. Anchorage-independent growth (AIG) was reduced by VPA. AIG was further reduced by combined treatment with irradiation. Expression of a stem cell marker, Oct4, was reduced by VPA. Oct4 was further decreased by combined treatment with irradiation. These results suggest that VPA may be a potential treatment for meningioma through targeting meningioma stem-like cells.

## INTRODUCTION

The presence of cancer stem cells (CSCs) is well documented in many kinds of human tumors [1]. With the ability of self-renewal [2], CSCs are considered responsible for tumor initiation, maintenance, and metastasis. Several studies have isolated human meningioma stem-like cells and characterized their CSCs properties. Isolated meningioma stem-like cells can form spheres and express the CD133 stem cell marker [3-5]. Moreover, isolated meningioma sphere cells (MgSCs) possess chemo- and radioresistant properties and express multiple drug-resistant genes, compared to meningioma adherent cells (MgACs) [3]. Studies by Kalamarides et al. have also demonstrated that different subtypes of meningioma come from a common prostaglandin D2 synthase-positive progenitor cell [6]. Although the biology of meningioma stem-like cells has been well established [3-6], therapeutic strategies targeting CSCs in meningioma remain unclear.

Meningioma is among the most common intracranial tumors and accounts for 13-26% of all intracranial neoplasms. The recurrence rate is reported to be 20% for World Health Organization (WHO) grade I, 40% for WHO grade II, and 78% for WHO grade III meningiomas [7]. Aggressive meningiomas are related to the high incidence of recurrence and mortality [8]. Management of recurrent meningioma currently is repeat surgical resection or stereotactic radiosurgery. Patients with aggressive meningiomas are treated with prior surgical resection followed by radiotherapy. However, atypical and anaplastic meningiomas remain challenging tumors to treat. Known risk factors such as a larger tumor size, nuclear atypia, increased mitotic rate, and necrosis are key to recurrence [8]. Since mainstream management of meningioma is surgical resection and stereotactic radiosurgery, anti-cancer drugs are less identified as treatment in management of meningioma. Without doubt, the development of adjuvant therapy is warranted to improve tumor control and minimize recurrence.

Valproic acid (VPA, 2-propylpentanoic acid) has been used extensively as an anti-convulsant for more than 40 years and is a frequent choice for patients with seizures [9]. As a histone deacetylase inhibitor, VPA can induce the differentiation of many kinds of cancer cells *in vitro* and suppress tumor growth and metastasis *in vivo* [10]. It improves the responsiveness of tumors to conventional therapeutic agents and increases the radiosensitivity of esophageal squamous cell carcinoma [11]. VPA has been investigated for its anti-cancer effect in many experimental human cancer models of lung cancer [12], renal cell carcinoma [13], bladder cancer [14], myeloma cell [15], and cervical cancer [16]. Its anti-tumor activity varies depending on the cell type and is conducted through multiple mechanisms, such as cell cycle arrest, apoptosis, angiogenesis, metastasis, differentiation, and senescence [17]. The purpose of this study is to examine the therapeutic potential of VPA through the targeting of MgSCs, and explore the related mechanisms.

## RESULTS

### Expression of Oct4 in MgSCs versus MgACs

**Figure 1:**
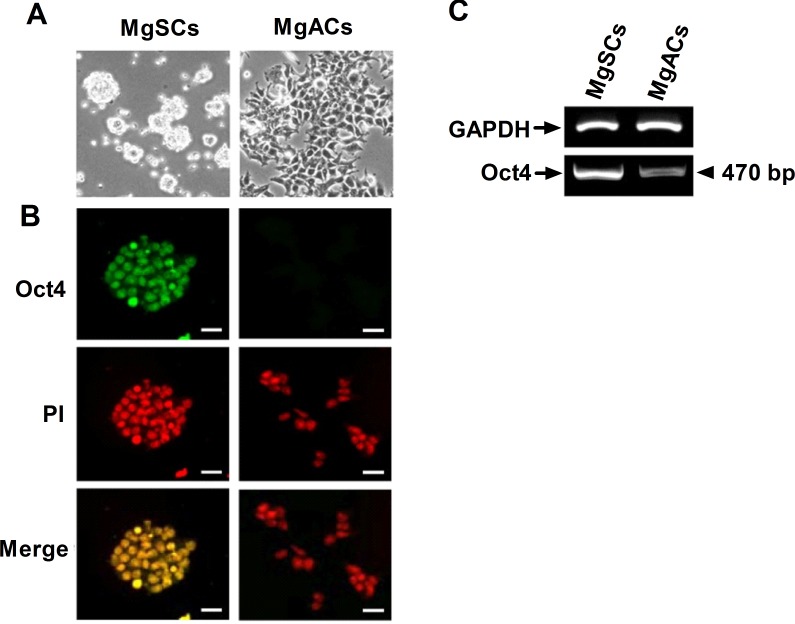
Expression of Oct4 in meningioma sphere cells (MgSCs) and meningioma adherent cells (MgACs) (**A**) Representative bright field micrographs showed growth of cultured MgSCs and MgACs in stem cell culture medium or DMEM with 10% FBS, respectively. (**B**) Oct4 expressions in MgSCs and MgACs were examined by immuno-fluorescence staining with anti-Oct4 antibody. Magnification, 100x. PI staining indicated the nucleus (red). Merged PI and Oct4 are shown in yellow. Bars: 100 μm. (**C**) Oct4 mRNA expression was examined by RT-PCR, with GAPDH as an internal control. Data are representative of 3 independent experiments.

To further characterize the stem-like properties of MgSCs, the expression of stem cell marker was analyzed by immunofluorescence and RT-PCR. Of the induced pluripotent stem (iPS) cell factors, Oct4 was first examined since it could reprogram adult stem cells to iPS cells as a single factor [18]. Day 2 cultured MgSCs formed spheres (Figure 1A) and were positive for Oct4 (Figure 1B), but daughter MgACs had relatively low expression on Oct4. Oct4 mRNA expressions in MgSCs and MgACs were determined by RT-PCR (Figure 1C).

### Cell viability of MgSCs and MgACs was reduced by VPA

**Figure 2:**
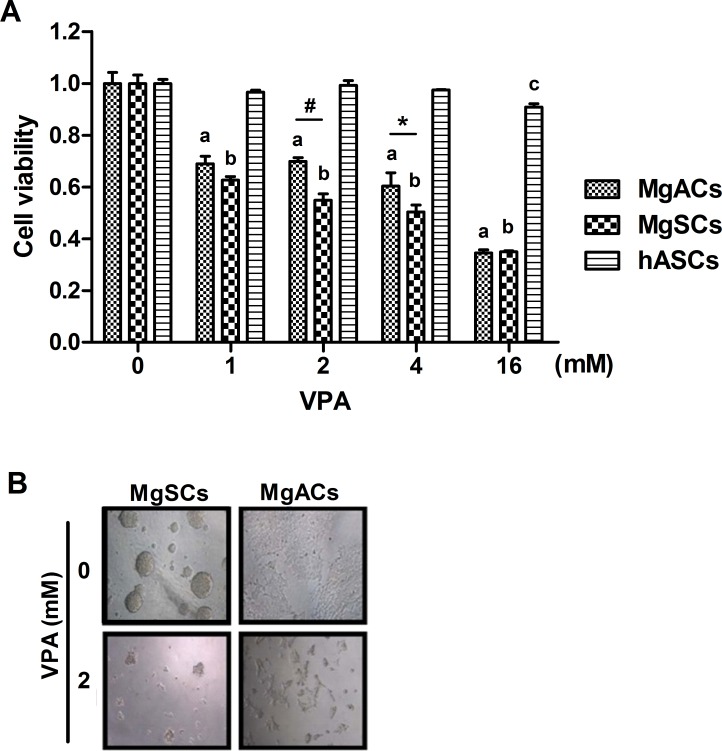
VPA decreased the cell viability of MgSCs and MgACs (**A**) MgSCs, MgACs, and hASCs were treated with 1, 2, 4, and 16 mM of VPA for 72 h, and cell viabilities were determined by MTS assay. (**B**) Bright field micrographs showed the cytotoxicity of VPA on MgSCs and MgACs. Bars, mean±SEM; a*, p* < 0.001 compared to MgACs without VPA; b, *p* < 0.001 compared to MgSCs without VPA; c*, p* < 0.05 compared to hASCs without VPA; *p < 0.05, ^#^*p* < 0.001 showed significant differences. Data are representative of 3 independent experiments.

VPA could induce apoptosis in tumors but not in non-malignant cells [19, 20]. To investigate cell toxicity of VPA on MgSCs and MgACs, cells were treated with VPA for 72 h and the cell viability was determined by MTS assay. At 1 mM ~16 mM, VPA reduced cell viability on both MgSCs and MgACs in a dose-response manner (*p*<0.001) (Figure 2A), but not on human adipocyte-derived stem cells (hASCs), which served as a non-malignant control, compared to untreated cells. MgSCs were more susceptible to VPA than MgACs at 2 and 4 mM. There were no differences in cell viability between MgSCs and MgACs at 16 mM VPA (Figure 2A). Representative microphotographs are shown in Figure 2B. These results showed that both MgSCs and MgACs were susceptible to VPA treatment, especially MgSCs.

### Combined treatment with VPA and irradiation induced cell cycle arrest, apoptosis, and DNA damage in MgSCs

**Figure 3:**
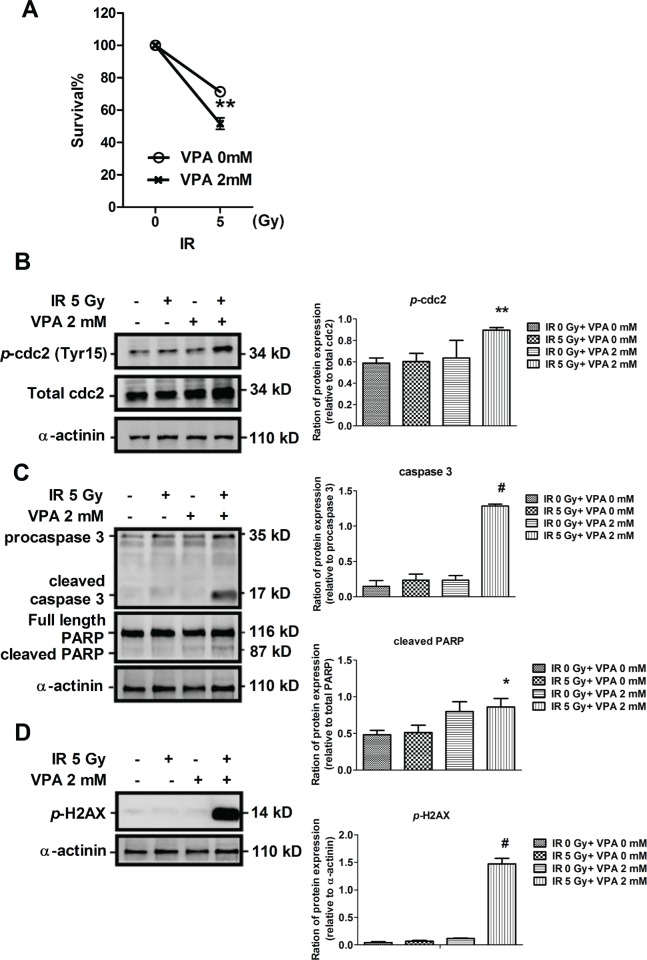
Effects of VPA and irradiation on cell viability and on cell cycle, apoptotic, and DNA damaging protein expressions in MgSCs MgSCs were treated with or without 2 mM VPA for 24 h followed by irradiation. (**A**) 24 h after irradiation, the cell viabilities were determined by MTS assay. Immuno-blots showed the (**B**) protein expressions of *p*-cdc2, (**C**) cleavage of caspase-3 and PARP, and (**D**) expression of *p*-H2AX of MgSCs treated with VPA and irradiation. α-actinin, loading control. The quantification results are shown in the right panel. Bars, mean±SEM; *p < 0.05, **p < 0.01, and ^#^*p* < 0.001 showed significant differences. Data are representative of 3 independent experiments.

Despite the chemoresistance of MgSCs to vincristine [3], VPA induced more severe cell death in MgSCs than in MgACs (Figure 2). The MgSCs were treated with an IC_50_ dose of VPA (2 mM) and irradiation (5Gy) alone or in combination, and were subjected to MTS assay. The results revealed that MgSCs pre-treated with VPA had reduced cell viability with the use of irradiation compared to the untreated control (*p* < 0.01) (Figure 3A), indicating that VPA increased the radio-sensitivity of MgSCs. Moreover, p-cdc2 (Tyr15), which was elevated in the G2/M phase of the cell cycle [21], was significantly induced by the combined treatment, compared to the untreated control (*p* < 0.01) (Figure 3B). The cleavage of apoptotic proteins, caspase-3 and PARP was also induced by the combined treatment (Figure 3C). DNA damage-inducing *p*-H2AX [22] was also significantly induced by the combined treatment, compared to the untreated control (*p* < 0.001) (Figure 3D). These findings indicate that VPA enhanced the radiosensitivity of MgSCs and that combined treatment decreased MgSCs survival through mechanisms like cell cycle arrest, apoptosis and DNA fragmentation.

### Combined VPA and irradiation decreased the colony formation of MgSCs

**Figure 4:**
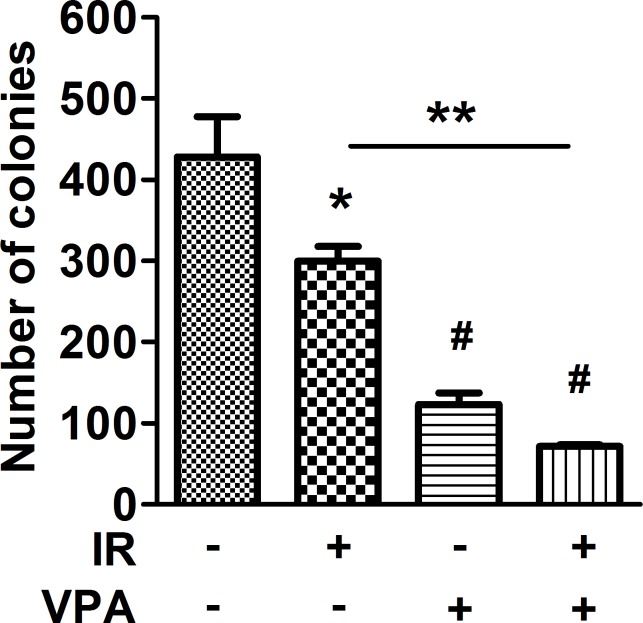
Effects of VPA and irradiation on colony formation of MgSCs MgSCs were treated with or without 2 mM VPA for 24 h, followed by irradiation 5 Gy. 24 h after irradiation, MgSCs were transferred and cultured in soft agar for 16 days. The colony formations were determined by soft agar assay and the quantitative results are shown. Bars, mean±SEM; *p < 0.05, **p < 0.01, and ^#^*p* < 0.001 showed significant differences. Data are representative of 3 independent experiments.

Since VPA could induce cell differentiation and anchorage-independent growth (AIG) was a key criterion for tumor metastasis [17], the effects of VPA and irradiation on MgSCs AIG were determined by soft-agar assay. The number of colony formations in MgSCs treated with radiation or VPA alone was significantly reduced (*p < 0.05 and ^#^*p* < 0.001, respectively) (Figure 4). Moreover, combined treatment significantly reduced colony formation compared to both the untreated control (^#^*p* < 0.001) and irradiation alone (**p < 0.01).

### Combined treatment with VPA and irradiation down-regulated Oct4 expression

**Figure 5:**
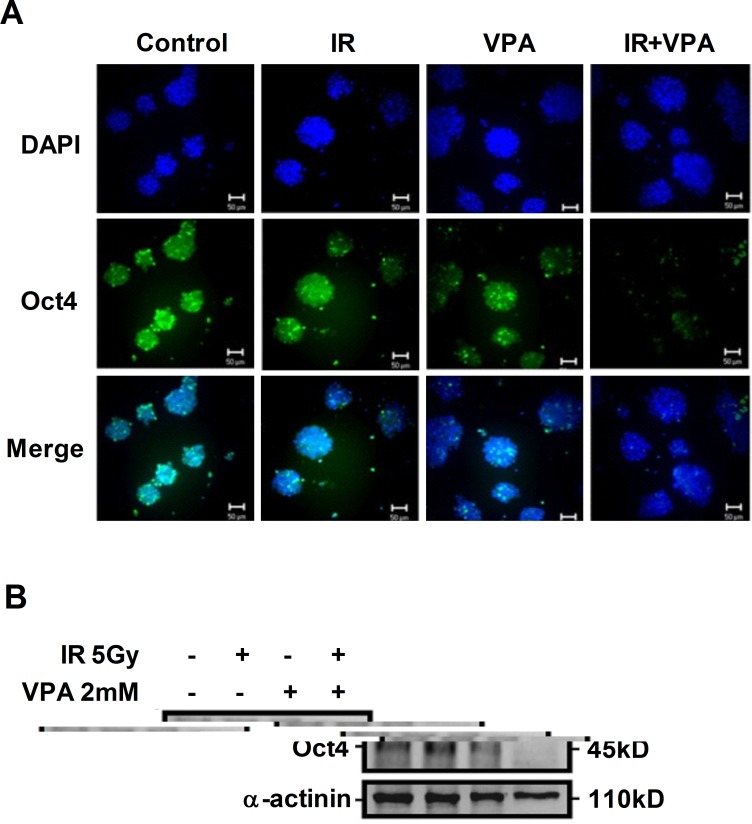
Effects of VPA and irradiation on Oct4 expression MgSCs were treated with or without 2 mM VPA for 24 h, followed by irradiation 5 Gy. 24 h after irradiation, the Oct4 expression was determined by (**A**) immuno-fluorescence and (**B**) immunoblotting. DAPI, nucleus; α-actinin, loading control. Scale bar: 50 μm.

To investigate the stem-like properties of MgSCs after treatment with VPA or irradiation alone or in combination, Oct4 expression was examined by immuno-fluorescence and immunoblotting. The expression of Oct4 in MgSCs was significantly reduced by VPA, but not by irradiation, compared to the untreated control (Figure 5). Combined treatment with VPA and irradiation further reduced the expression of Oct4 in MgSCs, compared to VPA alone.

## DISCUSSION

Using isolated meningioma stem-like cells, this study investigated the therapeutic potential of VPA and irradiation on drug-resistant meningioma. MgSCs treated with VPA prior to irradiation show reduced radio-resistance and anchorage-independent growth. Activation of signaling pathways, including apoptosis, cell cycle arrest, and DNA damage, were observed with combined treatment with VPA and irradiation, along with reduced Oct4 expression. These results suggest that combined treatment with VPA and irradiation might be a novel therapeutic strategy in the treatment of radioresistant meningiomas.

As CSCs are considered the major cause of chemo-resistance, radioresistance, and early recurrence of tumor, the induction of differentiation by VPA may hold promise in cancer therapy. Supporting this hypothesis, this study found that VPA reduces Oct4 expression in MgSCs (Figure 5). Moreover, AIG is greatly impaired by VPA treatment (Figure 4), suggesting the loss of stemness of MgSCs. The finding of the differentiation-promoting activity of VPA on MgSCs is consistent with previous studies on various cancer cells, including neuroblastoma [23], glioblastoma [24], head and neck cancer [25], thyroid cancer [26], and uveal melanoma [27]. Furthermore, recent studies using isolated glioblastoma-derived stem cells treated with VPA showed a reduced proliferation rate and expression of stem cell markers, including Oct4, indicating cell differentiation [28].

VPA also promotes cell self-renewal and re-programming in a cancer type-dependent manner. For example, inclusion of VPA in the reprogramming procedure can significantly increase the efficiency of iPS cells induction in cells over-expressing Oct4, Sox2, and Klf-4 [29]. Moreover, VPA increases the proliferation and self-renewal of normal hematopoietic stem cells (HSCs) to expand the HSC pool [30]. Mechanistic investigation by Teng et al. revealed that VPA activates the hormone response element on the Oct4 promoter through the PI3K/AKT/mTOR pathway and exhibits a pluripotency-promoting effect in myogenic cells [31, 32]. Studies on isolated breast cancer stem-like cells showed that VPA promotes cell de-differentiation via WNT/β-catenin signaling [33], suggesting the effect of VPA on CSCs was in a cancer type-dependent manner.

The divergent effect of VPA on cell differentiation has also been reported in previous studies, suggesting an association with cell differentiation level and underlying genetic alternation [34]. Since the functional proteomic expression varies with different cell types, the identification of specific molecular targets activated by VPA for promoting MgSCs differentiation may shed light on the application of VPA for cancer therapy.

The anti-cancer effects of VPA include cell cycle arrest, apoptosis, and DNA damage, and vary according to cell types [17]. In MgSCs, 2 mM VPA cannot significantly alter the activation of the apoptotic protein caspase-3 and PARP, cell cycle arrest protein *p*-cdc2, and DNA-damage inducing *p*-H2AX. However, the possibility that 2 mM VPA can induce cell death in MgSCs through cell apoptosis, cell cycle arrest, and DNA damage cannot be completely ruled out, since limited protein targets have been examined. In addition, previous studies have demonstrated that VPA also induces cell death through a caspase-independent pathway [35] and by autophagy [36]. Whether VPA induced MgSCs cell death through these pathways needs further investigation.

Combined treatment significantly induces *p*-cdc2, cleavage of caspase-3, cleavage of PARP, and *p*-H2AX expression with reduced cell viability, indicating a synergistic effect on signaling activation to promote cell death. VPA-induced radiosensitization has also been reported in various cancers, including colon cancer [37], esophageal squamous carcinoma [11], prostate cancer [38], and glioma [39]. With regard to combination therapies for CSCs, Jokinen et al. proposed drug ablation of the ALK oncogene using the ALK tyrosine kinase inhibitor, TAE684, in combination with PI3K inhibitor, or salinomycin. The features of CSCs were inverted with reduction of colony formation, indicating combination drug therapies can suppress CSCs features in acquired and adaptive resistance [40].

*In vivo* phase III clinical trials have been conducted or are ongoing using VPA alone or in combination with irradiation or other chemotherapeutic drugs for the treatment of cancers such as advanced cervical cancer (Table 1) [41]. Since VPA can pass the blood brain barrier, the above strong *in vivo* evidence from human clinical trials further supports the rationale for the potential application of VPA in the treatment of brain meningiomas. Our study clarifies the mechanism of action of VPA in meningioma stem-like cells, and further supports the evidence from the above *in vivo* clinical trials.

**Table 1:** Summary of ongoing, or completed clinical trials using valproic acid as a single agent or in combination with IR, or other agents

Cancer types	Phase of Trials	Serum level or dosage of VPA	Combined therapies	Outcome/Status	Reference/ClinicalTrials.gov Identifier
High Grade Gliomas and Brain Tumors	II	25 mg/kg/day, BID	TMZ + IR	Ongoing	NCT00302159^§^
Recurrent High-Grade Glioma	II	NA	Tosylate, Sildenafil Citrate	Recruiting	NCT01817751^§^
High Grade Gliomas	II	15 mg/kg/day	Bevacizumab	Recruiting	NCT00879437^§^
Brain Metastasis	I	20~50 mg/kg/day	TMZ + IR	NA	NCT00437957^§^
Refractory Solid or CNS Tumors	I	TID, trough level of 75 to 100 μg/mL	VPA as a single agent	Well tolerated	Su JM et al.[49]
Neuronal Tumors and Brain Metastases	I	10 mg/kg/day	Etoposide	NA	NCT00513162^§^

BID: Twice a day; NA: Not available; TID: three times daily; TMZ: Temozolomide; IR: irradiation

§ ClinicalTrials.gov Identifier number from website of https://clinicaltrials.gov/

Novel strategies based on identifying new mechanisms of old drugs may open new windows for developing chemotherapeutic agents targeting CSCs. Despite inducing CSCs differentiation, a novel concept targeting the conserved mitochondrial biogenesis pathway among CSCs to reduce its clonal expansion was also reported for FDA-approved antibiotics [42].

In summary, the present study suggests VPA is a potentially effective drug in the treatment of high recurrence meningioma. MgSCs and MgACs are both sensitive to VPA, which significantly reduces the radioresistance and anchorage-independent growth of MgSCs. VPA increases the susceptibility of MgSCs to irradiation. Oct4 expression in MgSCs was dramatically reduced by combined treatment with VPA and irradiation. These results also provide a novel insight into the development of an effective therapeutic strategy using a lower drug dosage and irradiation for the treatment of meningioma.

## MATERIALS AND METHODS

### Cell culture

The protocol for the maintenance of meningioma stem-like cells was as described previously [3]. In brief, meningioma stem-like cells were cultured in medium to obtain MgSCs from primary meningioma cells, while the control MgACs were maintained in Dulbecco modified Eagle medium (DMEM) with 10% fetal bovine serum (Harlan–Seralab, Belton, UK) on coating dishes.

Stem cell culture medium contained serum-free stem cell culture neurobasal DMEM/F12 medium (Gibco, CA, USA), B27 supplement, fresh aliquots of growth factors, 10 ng/ml recombinant human epidermal growth factor (EGF; Peprotech Rocky Hill, NJ, USA), and 10 ng/ml recombinant human basic fibroblast growth factor (bFGF; Peprotech, Rocky Hill, NJ, USA). The human adipose-derived stem cells (hASCs) were provided by S.M. Huang [43] and maintained in DMEM-low glucose (DMEM-LG; Invitrogen) with 10% fetal bovine serum (FBS; Invitrogen) and 1% penicillin-streptomycin (Invitrogen) to form sphere cells as non-tumor control cells. The cells were maintained in culture dishes at 37°C in 5% carbon dioxide.

The medium was changed every 3 days. When the cells grew to 70-90% confluence, they were trypsinized (0.25% trypsin; Sigma), and then neutralized by culture medium. The cells were passed at a ratio of 1:3. The characterization of human ASCs was as described previously [44].

### MTS assay

The CellTiter 96 Aqueous One Solution Cell Proliferation Assay kit (Promega, Madison, WI, USA) was used for MTS assay. MgSCs, MgACs, and hASCs (2×10^4^) were grown in 96-well plates with fresh culture medium. Following treatment with control solvent or VPA, 20 μl MTS solution was added to each well and the plate was allowed to incubate for 4 h at 37°C. The same dilution of MTS solution in DMEM/F12 medium alone was used as the background.

After incubation, absorbance was recorded at 490 nm. For data analysis, background values were subtracted from all sample values. The calculated absorbance was directly proportional to the number of living cells in the culture.

### Reagents and irradiation treatment

VPA was obtained from Sanofi-Aventis (Paris, France). Antibody against Oct4, *p*-cdc2, total cdc2, and PARP were purchased from Cell Signaling (Danvers, MA). Antibody against caspase-3 and *p*-H2AX were purchased from Abcam, Inc. (Cambridge, MA). Antibody against α -actinin was purchased from Santa Cruz Biotech, Inc. (Santa Cruz, CA). For the radioresistance assay, the cells were irradiated using a CyberKnife radio-surgery system (Accuray, USA) to deliver different doses.

### Immunofluorescence staining

The immunofluorescence staining followed the previous method [3, 21, 45, 46]. The MgACs and MgSCs were fixed using 2% paraformaldehyde (PFA) in phosphate-buffered saline (PBS) for 20 min, blocked with bovine serum albumin for 1 h, washed with PBS containing 0.1% Tween 20, and stained with Oct4 antibody, nuclear dye propidium iodide (PI) (Sigma, St Louis, MO, USA), or nuclear dye 4′,6-diamidino-2-phenylindole (DAPI) for 1 h at 4°C. Immunofluorescence was detected after incubation with the appropriate secondary antibodies conjugated with FITC (eBioscience, San Diego, CA, USA) at room temperature for 45 min. After mounting cover-slips with SlowFade Light anti-fade reagent (Molecular Probes, Eugene, OR, USA), immunofluorescent pictures were acquired using a CCD camera (Zeiss). The scale bar was labeled using the SPOT RT3 software (Diagnostic Instruments, Sterling Heights, MI, USA).

### Cell lysate preparation and Western blots

Cells were harvested by centrifugation at 1000g for 10 min and lysed by RIPA buffer (100mM Tris-HCl, 150mM NaCl, 0.1% SDS, and 1% Triton-X-100) at 4°C for 10 min. The cell lysates were harvested by centrifugation at 15000 rpm for 10 min to obtain the supernatants for Western blotting. In brief, aliquots of 20 μg proteins from each group were applied to 10% sodium dodecyl sulfate polyacrylamide gels and electrophoresed for 3 h at 80 V. Proteins were transferred onto polyvinyldifluoride membranes (Millipore) and blocked with 5% bovine serum albumin in PBS for 2 h at room temperature. Band detection was conducted by enhanced chemiluminescence (Millipore) and an LAS-3000 imaging system (Fujifilm, Tokyo, Japan). Band densities were measured with the gel analysis system (BioSpectrumAC Imaging System Vision Work LS software; UVP, Upland, CA, USA) [47].

### RNA isolation and RT-PCR

Total RNA was extracted using EasyPure Total RNA reagent (Bioman, Taiwan, ROC) according to the manufacturer's instructions. Total RNA (1.0 μg) was reverse transcribed (RT) with MMLV ReverseTranscriptase (Epicentre Biotechnologies, USA) according to the manufacturer's instructions. The primer pairs used were: Oct4 forward, 5′-GAGAATTTGTTCCTGCAGTGC-3′ and reverse, 5′-GTTCCCAATTCCTTCCTTAGTG-3′ [48] and GAPDH forward, 5′-CTTCATTGACCTCAACTAC-3′ and reverse, 5′-GCCATCCACAGTCTTCTG-3′. The PCR products were subjected to 1.5% agarose gel and visualized with UV light after ethidium bromide staining.

### Anchorage-independent growth in soft agar assay

Cell suspensions (1×10^4^) were incubated in an upper layer of 0.3% agar (Difco, Detroit, MI, USA) in DMEM with 2% FBS. This was overlaid on 0.5% basal agar with 2% FBS. Cultures were maintained for 16 days, replenishing the upper medium layer twice a week, and then staining with methylene blue diluted in ethanol. Colonies were counted by microscopy (CK2; Olympus, Tokyo, Japan).

### Statistical analysis

All data were calculated using IBM SPSS statistics 20. Data were expressed as mean±standard error of the mean (SEM) and differences between counts were determined using One way Anova. Statistical significance was set at *p* < 0.05.

## References

[1] Jordan CT, Guzman ML, Noble M (2006). Cancer stem cells. The New England Journal of Medicine.

[2] Gutmann DH (2014). The taxonomy of brain cancer stem cells: what's in a name?. Oncoscience.

[3] Hueng DY, Sytwu HK, Huang SM, Chang C, Ma HI (2011). Isolation and characterization of tumor stem-like cells from human meningiomas. Journal of Neuro-Oncology.

[4] Tang H, Gong Y, Mao Y, Xie Q, Zheng M, Wang D, Zhu H, Wang X, Chen H, Chen X, Zhou L (2012). CD133-positive cells might be responsible for efficient proliferation of human meningioma cells. International Journal of Molecular Sciences.

[5] Rath P, Miller DC, Litofsky NS, Anthony DC, Feng Q, Franklin C, Pei L, Free A, Liu J, Ren M, Kirk MD, Shi H (2011). Isolation and characterization of a population of stem-like progenitor cells from an atypical meningioma. Experimental and Molecular Pathology.

[6] Kalamarides M, Stemmer-Rachamimov AO, Niwa-Kawakita M, Chareyre F, Taranchon E, Han ZY, Martinelli C, Lusis EA, Hegedus B, Gutmann DH, Giovannini M (2011). Identification of a progenitor cell of origin capable of generating diverse meningioma histological subtypes. Oncogene.

[7] Yoo-Jin K, Kim Y, Bochem N, Ketter R, Henn W, Feiden W (2008). Meningiomas: multiparametric approach for risk stratification and grading. Der Pathologe.

[8] Ferraro DJ, Funk RK, Blackett JW, Ju MR, Dewees TA, Chicoine MR, Dowling JL, Rich KM, Drzymala RE, Zoberi I, Simpson JR, Jaboin JJ (2014). A retrospective analysis of survival and prognostic factors after stereotactic radiosurgery for aggressive meningiomas. Radiat Oncol.

[9] van Breemen MS, Rijsman RM, Taphoorn MJ, Walchenbach R, Zwinkels H, Vecht CJ (2009). Efficacy of anti-epileptic drugs in patients with gliomas and seizures. Journal of Neurology.

[10] Gottlicher M, Minucci S, Zhu P, Kramer OH, Schimpf A, Giavara S, Sleeman JP, Lo Coco F, Nervi C, Pelicci PG, Heinzel T (2001). Valproic acid defines a novel class of HDAC inhibitors inducing differentiation of transformed cells. The EMBO Journal.

[11] Shoji M, Ninomiya I, Makino I, Kinoshita J, Nakamura K, Oyama K, Nakagawara H, Fujita H, Tajima H, Takamura H, Kitagawa H, Fushida S, Harada S, Fujimura T, Ohta T (2012). Valproic acid, a histone deacetylase inhibitor, enhances radiosensitivity in esophageal squamous cell carcinoma. International Journal of Oncology.

[12] Liu X, Chen L, Sun F, Zhang G (2013). Enhanced suppression of proliferation and migration in highly-metastatic lung cancer cells by combination of valproic acid and coumarin-3-carboxylic acid and its molecular mechanisms of action. Cytotechnology.

[13] Yang FQ, Liu M, Yang FP, Che J, Li W, Zhai W, Wang GC, Zheng JH, Li X (2014). VPA inhibits renal cancer cell migration by targeting HDAC2 and down-regulating HIF-1alpha. Molecular Biology Reports.

[14] Wang D, Jing Y, Ouyang S, Liu B, Zhu T, Niu H, Tian Y (2013). Inhibitory effect of valproic acid on bladder cancer in combination with chemotherapeutic agents and. Oncology Letters.

[15] Moreaux J, Reme T, Leonard W, Veyrune JL, Requirand G, Goldschmidt H, Hose D, Klein B (2013). Gene expression-based prediction of myeloma cell sensitivity to histone deacetylase inhibitors. British Journal of Cancer.

[16] Han BR, You BR, Park WH (2013). Valproic acid inhibits the growth of HeLa cervical cancer cells via caspase-dependent apoptosis. Oncology Reports.

[17] Duenas-Gonzalez A, Candelaria M, Perez-Plascencia C, Perez-Cardenas E, de la Cruz-Hernandez E, Herrera LA (2008). Valproic acid as epigenetic cancer drug: preclinical, clinical and transcriptional effects on solid tumors. Cancer Treatment Reviews.

[18] Kim JB, Greber B, Arauzo-Bravo MJ, Meyer J, Park KI, Zaehres H, Scholer HR (2009). Direct reprogramming of human neural stem cells by OCT4. Nature.

[19] Nebbioso A, Clarke N, Voltz E, Germain E, Ambrosino C, Bontempo P, Alvarez R, Schiavone EM, Ferrara F, Bresciani F, Weisz A, de Lera AR, Gronemeyer H, Altucci L (2005). Tumor-selective action of HDAC inhibitors involves TRAIL induction in acute myeloid leukemia cells. Nature Medicine.

[20] Insinga A, Monestiroli S, Ronzoni S, Gelmetti V, Marchesi F, Viale A, Altucci L, Nervi C, Minucci S, Pelicci PG (2005). Inhibitors of histone deacetylases induce tumor-selective apoptosis through activation of the death receptor pathway. Nature Medicine.

[21] Ma HI, Chiou SH, Hueng DY, Tai LK, Huang PI, Kao CL, Chen YW, Sytwu HK (2011). Celecoxib and radioresistant glioblastoma-derived CD133+ cells: improvement in radiotherapeutic effects. Laboratory investigation. Journal of Neurosurgery.

[22] Sharma A, Singh K, Almasan A (2012). Histone H2AX phosphorylation: a marker for DNA damage. Methods Mol Biol.

[23] Cinatl J, Jr., Cinatl J, Scholz M, Driever PH, Henrich D, Kabickova H, Vogel JU, Doerr HW, Kornhuber B (1996). Antitumor activity of sodium valproate in cultures of human neuroblastoma cells. Anti-cancer Drugs.

[24] Roy Choudhury S, Karmakar S, Banik NL, Ray SK (2011). Valproic acid induced differentiation and potentiated efficacy of taxol and nanotaxol for controlling growth of human glioblastoma LN18 and T98G cells. Neurochemical Research.

[25] Gan CP, Hamid S, Hor SY, Zain RB, Ismail SM, Wan Mustafa WM, Teo SH, Saunders N, Cheong SC (2012). Valproic acid: growth inhibition of head and neck cancer by induction of terminal differentiation and senescence. Head & Neck.

[26] Haghpanah V, Malehmir M, Larijani B, Ahmadian S, Alimoghaddam K, Heshmat R, Ghavamzadeh A, Adabi K, Ghaffari SH (2014). The Beneficial Effects of Valproic Acid in Thyroid Cancer Are Mediated through Promoting Redifferentiation and Reducing Stemness Level: An *In Vitro* Study. Journal of Thyroid Research.

[27] Landreville S, Agapova OA, Matatall KA, Kneass ZT, Onken MD, Lee RS, Bowcock AM, Harbour JW (2012). Histone deacetylase inhibitors induce growth arrest and differentiation in uveal melanoma. Clinical cancer research: an official journal of the American Association for Cancer Research.

[28] Alvarez AA, Field M, Bushnev S, Longo MS, Sugaya K (2015). The Effects of Histone Deacetylase Inhibitors on Glioblastoma-Derived Stem Cells. Journal of Molecular Neuroscience.

[29] Huangfu D, Maehr R, Guo W, Eijkelenboom A, Snitow M, Chen AE, Melton DA (2008). Induction of pluripotent stem cells by defined factors is greatly improved by small-molecule compounds. Nature Biotechnology.

[30] Bug G, Gul H, Schwarz K, Pfeifer H, Kampfmann M, Zheng X, Beissert T, Boehrer S, Hoelzer D, Ottmann OG, Ruthardt M (2005). Valproic acid stimulates proliferation and self-renewal of hematopoietic stem cells. Cancer Research.

[31] Teng HF, Kuo YL, Loo MR, Li CL, Chu TW, Suo H, Liu HS, Lin KH, Chen SL (2010). Valproic acid enhances Oct4 promoter activity in myogenic cells. Journal of Cellular Biochemistry.

[32] Teng HF, Li PN, Hou DR, Liu SW, Lin CT, Loo MR, Kao CH, Lin KH, Chen SL (2014). Valproic acid enhances Oct4 promoter activity through PI3K/Akt/mTOR pathway activated nuclear receptors. Molecular and Cellular Endocrinology.

[33] Debeb BG, Lacerda L, Xu W, Larson R, Solley T, Atkinson R, Sulman EP, Ueno NT, Krishnamurthy S, Reuben JM, Buchholz TA, Woodward WA (2012). Histone deacetylase inhibitors stimulate dedifferentiation of human breast cancer cells through WNT/beta-catenin signaling. Stem Cells.

[34] Kuendgen A, Gattermann N (2007). Valproic acid for the treatment of myeloid malignancies. Cancer.

[35] Kawagoe R, Kawagoe H, Sano K (2002). Valproic acid induces apoptosis in human leukemia cells by stimulating both caspase-dependent and -independent apoptotic signaling pathways. Leukemia Research.

[36] Fu J, Shao CJ, Chen FR, Ng HK, Chen ZP (2010). Autophagy induced by valproic acid is associated with oxidative stress in glioma cell lines. Neuro-Oncology.

[37] Chen X, Wong P, Radany E, Wong JY (2009). HDAC inhibitor, valproic acid, induces p53-dependent radiosensitization of colon cancer cells. Cancer Biotherapy & Radiopharmaceuticals.

[38] Chen X, Wong JY, Wong P, Radany EH (2011). Low-dose valproic acid enhances radiosensitivity of prostate cancer through acetylated p53-dependent modulation of mitochondrial membrane potential and apoptosis. Molecular Cancer Research: MCR.

[39] Zhou Y, Xu Y, Wang H, Niu J, Hou H, Jiang Y (2014). Histone deacetylase inhibitor, valproic acid, radiosensitizes the C6 glioma cell line. Oncology Letters.

[40] Jokinen E, Laurila N, Koivunen P, Koivunen JP (2014). Combining targeted drugs to overcome and prevent resistance of solid cancers with some stem-like cell features. Oncotarget.

[41] Coronel J, Cetina L, Pacheco I, Trejo-Becerril C, Gonzalez-Fierro A, de la Cruz-Hernandez E, Perez-Cardenas E, Taja-Chayeb L, Arias-Bofill D, Candelaria M, Vidal S, Duenas-Gonzalez A (2011). A double-blind, placebo-controlled, randomized phase III trial of chemotherapy plus epigenetic therapy with hydralazine valproate for advanced cervical cancer. Preliminary results. Med Oncol.

[42] Lamb R, Ozsvari B, Lisanti CL, Tanowitz HB, Howell A, Martinez-Outschoorn UE, Sotgia F, Lisanti MP (2015). Antibiotics that target mitochondria effectively eradicate cancer stem cells, across multiple tumor types: Treating cancer like an infectious disease. Oncotarget.

[43] Huang SP, Huang CH, Shyu JF, Lee HS, Chen SG, Chan JY, Huang SM (2013). Promotion of wound healing using adipose-derived stem cells in radiation ulcer of a rat model. Journal of Biomedical Science.

[44] Huang SP, Hsu CC, Chang SC, Wang CH, Deng SC, Dai NT, Chen TM, Chan JY, Chen SG, Huang SM (2012). Adipose-derived stem cells seeded on acellular dermal matrix grafts enhance wound healing in a murine model of a full-thickness defect. Annals of Plastic Surgery.

[45] Lee CC, Jan HJ, Lai JH, Ma HI, Hueng DY, Lee YC, Cheng YY, Liu LW, Wei HW, Lee HM (2010). Nodal promotes growth and invasion in human gliomas. Oncogene.

[46] Tsai WC, Chen Y, Huang LC, Lee HS, Ma HI, Huang SM, Sytwu HK, Hueng DY (2013). EMMPRIN expression positively correlates with WHO grades of astrocytomas and meningiomas. Journal of Neuro-Oncology.

[47] Hueng DY, Lin GJ, Huang SH, Liu LW, Ju DT, Chen YW, Sytwu HK, Chang C, Huang SM, Yeh YS, Lee HM, Ma HI (2011). Inhibition of Nodal suppresses angiogenesis and growth of human gliomas. Journal of Neuro-Oncology.

[48] Amini S, Fathi F, Mobalegi J, Sofimajidpour H, Ghadimi T (2014). The expressions of stem cell markers: Oct4, Nanog, Sox2, nucleostemin, Bmi, Zfx, Tcl1, Tbx3, Dppa4, and Esrrb in bladder, colon, and prostate cancer, and certain cancer cell lines. Anatomy & Cell Biology.

[49] Su JM, Li XN, Thompson P, Ou CN, Ingle AM, Russell H, Lau CC, Adamson PC, Blaney SM (2011). Phase 1 study of valproic acid in pediatric patients with refractory solid or CNS tumors: a children's oncology group report. Clinical cancer research: an official journal of the American Association for Cancer Research.

